# AD Biomarker NPTX2 Loss of Function Drives Sleep and Circadian Rhythm Disruption

**DOI:** 10.1002/alz70855_103059

**Published:** 2025-12-23

**Authors:** Seung‐Eon Roh, Mei‐Fang Xiao, Kathryn Cheong, Chuljung Kwak, Ana Delgado, Alena Savonenko, Arnold Bakker, Douglas R. Galasko, Hyung‐Bae Kwon, Paul F Worley

**Affiliations:** ^1^ Johns Hopkins University School of Medicine, Baltimore, MD, USA; ^2^ Institute for Basic Science, Daejeon, Chungcheong, Korea, Republic of (South); ^3^ Johns Hopkins University, Baltimore, MD, USA; ^4^ Department of Psychiatry and Behavioral Science, Johns Hopkins University School of Medicine, Baltimore, MD, USA; ^5^ Department of Cellular and Molecular Medicine, University of California, San Diego, La Jolla, CA, USA; ^6^ Shiley‐Marcos Alzheimer's Disease Research Center, University of California, San Diego, CA, USA

## Abstract

**Background:**

Sleep and circadian rhythm disruption (SCRD) is prevalent in aging, particularly in individuals transitioning to mild cognitive impairment (MCI) and Alzheimer's disease (AD). Orexin A, a key neuropeptide regulating wakefulness, is reduced in conditions like narcolepsy. The synaptic immediate early gene NPTX2, co‐expressed in orexin neurons, serves as a sensitive biomarker for AD and has been linked to cognitive decline. The precise role of NPTX2 in SCRD, however, remains unclear.

**Method:**

NPTX2 and orexin A levels in cerebrospinal fluid (CSF) were analyzed in cognitively normal aged individuals and AD patients across two independent cohorts. In NPTX2 knockout (KO) mice, orexin A levels were measured using biochemical assays. Behavioral assessments and sleep EEG recordings were performed to investigate the contribution of NPTX2 to SCRD, focusing on circadian onset, vigilance states, sleep transitions, orexin signaling, neuronal synchronicity, and sleep spindle activity.

**Result:**

NPTX2 levels in CSF strongly correlated with orexin A in cognitively normal aged individuals but not in AD, suggesting a disruption in this association with disease progression. However, orexin A expression was not altered in NPTX2 KO and they exhibited disrupted circadian onset, increased activity during the sleep phase, and fragmented sleep characterized by increased wake‐to‐NREM transitions and altered vigilance state durations. Behavioral assays demonstrated an enhanced response to the orexin receptor 1 agonist TAK‐925 in NPTX2 KO mice, indicating altered orexin signaling. Sleep EEG analysis revealed shifts in power across frequency bands and reduced sleep spindles, an early marker of human sleep disruption.

**Conclusion:**

These findings demonstrate that NPTX2 loss of function instigates SCRD by disrupting sleep architecture, orexin signaling, and neuronal synchronicity. This supports the effector role of NPTX2 in sleep regulation and highlights its potential as a therapeutic target for sleep and circadian dysfunction in aging and neurodegenerative conditions.

